# Explicit measures for emotional congruence with children are related to sexual interests in a male community sample, but not implicit measures

**DOI:** 10.1038/s41443-024-00911-9

**Published:** 2024-06-20

**Authors:** Dahlnym Yoon, Miriam J. Hofmann, Andreas Mokros, Jonas Krüppel

**Affiliations:** 1 Institute for Forensic Psychology and Forensic Medicine (IFPM), MSH – Medical School Hamburg, Hamburg, Germany; 2 Faculty of Psychology, FernUniversität in Hagen (University of Hagen), Hagen, Germany

**Keywords:** Risk factors, Diagnosis

## Abstract

This study sought to disentangle several phenotypic correlates of pedophilic sexual interests, such as emotional congruence with children and lack of empathy. We utilized Implicit Association Tests and self-report questionnaires for emotional congruence with children and analyzed the psychometric properties of these measures. Further, we analyzed the associations between these measures and self-reported pedophilic sexual interests and empathy. The sample consisted of 110 adult community males (prevalence of sexual interest in children at least to some extent: 5.5%) with or without child-related jobs (43.6% vs. 56.4%) recruited online in the general population. Overall, we found equivocal parameters for reliability of the implicit and explicit measures of emotional congruence with children (α = 0.29 to 0.76). The self-reported emotional congruence with children was only weakly linked to the implicit positive evaluations of children (*r* = 0.170, *p* = 0.039), not linked to empathy except for distress in social interactions (*r* = 0.199, *p* = 0.019), and moderately linked to deviant sexual interests in children (*r* = 0.321 to 0.404, *p* < 0.001), especially in men working with children (*R*^2^ = 0.04, *p* = 0.027). Further studies are warranted to expand the sample to the offender population and explore the more complex network of constructs related to pedophilia.

## Introduction

Pedophilia is one of the most important risk factors for recurrent sexual offenses against children and thus an important treatment target [1–3]. Prevalence estimates of pedophilic interests vary from 4.10% up to 9.50% in the male population, whereas those of pedophilic disorders varies from 0.23% to 0.60% [4–7]. The estimated prevalence of the pedophilic disorder in males convicted of sexual crimes varies even more vastly depending on the sample. Whereas the prevalence reports from North-America ranged from 12% in so-called “dangerous” offenders with child sexual victims [8] to around 50% in sexual offenders in general [9], the range was higher in German-speaking countries from 27% in child sexual abusers including child sexual exploitation materials abusers (*N* = 304; [10]) to 67.1% in contact child sexual abusers (*n* = 671; [11]). Previous research has also indicated that child sexual abusers working with children show stronger pedophilic interests than other child sexual abusers (*F* = 40.713, *p* < 0.001; [12]).

Assessment of pedophilic interests and phenomenological correlates faces two major challenges: First, there is often only moderate agreement between different methods for detecting pedophilic interests [13, 14]. This might be due to the circumstance that the most common explicit measures, such as questionnaires and structured interviews, rely on self-reports of an individual. Discrepancies in the appraisal of deviant sexual interests could occur due to a possible lack of ability or willingness to accurately describe oneself, or both [15]. Apart from a lack of ability to reflect and verbalize one’s own sexuality, fears of stigma could also hinder the willingness to provide information required for an accurate assessment [16]. In addition, the core criteria of pedophilia, e.g., sexual fantasies, sexual interests, or constructs that are phenomenologically associated with pedophilia, e.g., emotional congruence with children (ECWC), empathy, or cognitive distortions [17] might lie at the subconscious level of cognition.

The second challenge in assessment is the scarcity of well-validated instruments that are able to capture the constructs related to pedophilic interests [18]. An individual etiology of one’s sexual offense requires a more sophisticated understanding of constructs related to pedophilic interests. Such explanatory hypotheses could help improving risk appraisals, but also setting treatment targets based on those. Availability of instruments to measure these constructs can thus impact not only on the accuracy of the diagnostic classification and risk assessments, but also on the treatment of those affected.

Since both pedophilia and its phenotypic correlates are linked to recidivism and treatment progress to varying degrees [19], an adequate assessment and differentiation of these constructs are essential. Against this backdrop and the above-mentioned challenges, an empirically based differentiation of these phenomenological correlates of pedophilia has been demanding.

### Emotional congruence in etiological models of sexual abuse

Research has found emotional immaturity and distorted emotional processing in men with pedophilia [20–23]. It is often observed that people suffering from pedophilia report having “fallen in love” with a child and find gratification from spending time with or thinking about the child(ren) [3, 24]. According to Finkelhor [25], this ECWC is a construct that must be distinguished from sexual interest in children, since not all people with a special emotional connection to children necessarily experience sexual arousal (e.g., as parents, teachers, or pediatricians).

#### Validity of the emotional congruence construct

According to the meta-analysis by Hanson and Morton-Bourgon [2], ECWC is a significant predictor of recidivism (*k* = 3*, n* = 3419*, d* = 0.42). Similar results were provided in the meta-analysis by McPhail et al. [26], especially for child abusers with extrafamilial victims (*k* = 5*, n* = 639*, d* = 0.5). ECWC was also significantly increased in this group compared to those with intrafamilial victims (*k* = 15*, n* = 3928*, d* = 0.54). Since the publication of these meta-analyses, only a handful of studies have been published that dealt with the predictive validity of ECWC.

Hermann et al. [27] found in 424 male sexual offenders that pedophilc interests can distinguish men with low vs. high ECWC (*AUC* = 0.615). In another recent study by Konrad et al. [28], 217 non-offending pedophilic men in the “Dunkelfeld” project and 22 non-offending non-pedophilic men could be discriminated by the ECWC construct regarding their diagnoses, *F*(2, 236) = 5.78*, p* = 0.004.

#### Emotional congruence in contrast to empathy

Cuff et al. [29] found 43 definitions of empathy, which revolve around the various debates, such as cognitive vs. affective components in the construct, correspondence between the perceived and evoked emotion, traits and states influences, and automatic vs. controlled process. In closer look, the description of increasing empathy as a treatment goal in forensic context lean into general perspective taking [3, 30]. Mann et al. [3] further pointed out that a lack of victim empathy could be a manifestation of justifications on the part of the perpetrators, but also a general affective deficit measured with Hare’s Psychopathy Checklist-Revised [31], which in turn represents an empirically proven predictor for relevant recidivism in sexual offenders (e.g., *d* = 29, [2]; *AUC* = 0.60*−*0.65; [31, 32]).

Although ECWC appears to be both etiologically and prognostically relevant, treatment programs for people who committed sexual offenses against children focus rather on empathy than emotional congruence [30]. Empathy and ECWC can be differentiated in terms of deficits in empathy being a positive misattribution of joy and denial of negative feelings of children, whereas ECWC being positive emotional experiences in contact with children, as well as desire and preference of close relationships with children than adults [17, 33], which goes beyond an empathic ability per se. Empathy would rather depict the lack of perspective taking, cold-heartedness, and lack of emotional concern about others even in close relationships (STABLE-2007, cf. section: *Explicit Measures* of ECWC; [24]). A person with a high level of empathy could perceive children’s emotions well and process them affectively without sexually preferring them; at the same time, a person with ECWC might misidentify or ignore the child’s negative emotions. Whether this misidentification is rather a deficit in empathic capacity or a consequence of ECWC is debatable [17]. ﻿A﻿ clearer demarcation of constructs could improve (a) measuring the respective constructs more reliably or selectively, (b) determining the validity of these constructs for behavioral outcomes – such as recidivism –, and (c) examining changes of these constructs during treatment more reliably.

#### Measures of ECWC

##### Explicit measures

The STABLE-2007 [24] for the clinical assessment of dynamic risk factors in sexual offenders operationalizes ECWC as emotional identification with children, preference of contact with children rather than with adults, as well as age-inappropriate interests and activities.

There are several self-report questionnaires available, for instance, the *Emotional Congruence with children scale–Children and Sex Questionnaire* (EC–CSQ; [34]) and the *Child Identification Scale-Revised* (CIS-R; [35]). Both procedures – to our knowledge – have been neither translated nor validated in German-speaking countries. Overall, these questionnaires showed heterogeneous psychometric properties regarding factor structure [26, 28, 35], reliability and criterion-related validity [26, 28]. Next to the unsatisfying psychometric properties of the questionnaires for ECWC, Paquette and Cortoni [36] also criticized the outdated concentration on contact sexual offenses, since online sexual exploitation or usage of materials depicting child sexual exploitation have been suggested as a strong indicator for sexual interests in children. Thus, a recently developed *Cognitive and Emotional Congruence With Children scale* (C-ECWC; [33]) was implemented in the current study (see method section for a detailed description of the questionnaire).

##### Implicit measures

The Implicit Association Test (IAT; [37]) is one of the implicit measures that are well-implemented in the assessment of deviant sexual interests. In the IAT, the cross-association between two different targets (e.g., flowers vs. insects) and two opposing attributes (e.g., good vs. bad) is estimated based on reaction time differences in compatible and incompatible combinations of both. The IAT has been established as a measure with good psychometric properties in sexual offender assessment [38], even if its underlying mechanism is still debated [39–41]. In a sample of 25 sexual offenders, 22 non-sexual offenders, and 54 students, McPhail et al. [42] found small but not significant correlations between the childlike self-identification (Identification IAT) and the EC-CSQ and CIS-R (*r* = 0.25 and *r* = 0.10, *p* > 0.05, respectively), as well as a moderate significant correlation between the positive attribution towards children (Evaluation IAT) and the EC-CSQ (*r* = 0.46*, p* < 0.05), and a moderate but not significant correlation between the Evaluation IAT and the CIS-R (*r* = 0.35, *p* > 0.05). Both IATs were rather moderately to strongly related to professional assessments of recidivism risks or pedophilia (*r* = *−*0.34, *p* > 0.05 to *r* = 0.58, *p* < 0.01), but the sample sizes were very small for each correlation (*n* = 10 to 19). The relationship between the ECWC questionnaires and clinically diagnosed pedophilia was inconclusive (*r* = 0.33, *p* > 0.05 for the CIS-R and *r* = 0.60, *p* < 0.01 for the EC-CSQ). Compared to professional assessment tools, self-report questionnaires are clearly more economical, but the above-mentioned criticism regarding desirable responding or lack of introspective capacity applies to these measures more clearly than to the professional assessment or other informant ratings. Thus, the potential usefulness of implicit measures has been emphasized and multi-method approaches that combine both implicit and explicit approaches appear to be promising in the field of assessment of pedophilia and its related constructs [13]. In this article, the term implicit will be used as an overarching term for a class of structurally similar instruments (including the IAT), irrespective of the underlying cognitive process [39–41].

#### Research questions

The present study is a part of an on-going project on the measurement of phenomenological correlates of pedophilia, in which we attempt to differentiate these constructs. As the first step of the project, we conducted the present study using the implicit and explicit measures to assess ECWC, replicating and expanding the findings by McPhail et al. [42] and Paquette and McPhail [33] in a male community sample, given these studies were also conducted in male samples. The pre-registration of the current study is available under: https://osf.io/2sf74/.

#### RQ1: validity of the implicit and explicit measures of ECWC

The first part of the study intended to cross-validate both the evaluation and the identification IATs developed by McPhail et al. [43] and the C-ECWC scale by Paquette and McPhail [33]. We first hypothesized positive relationships between implicitly and explicitly measured ECWC (H1.1), between implicit ECWC and explicit empathy (H1.2), and lastly (H1.3), that the construct immanent correlations of the ECWC measures (H1.1) are stronger than those between implicit ECWC and explicit empathy (H1.2).

#### RQ2: construct validity of ECWC

Assuming that both implicit and explicit measures of ECWC are valid, we expected that we can establish construct validity of ECWC using these measures. Thus, we hypothesized differential links of these measures to external correlates. We assumed that implicitly and explicitly measured ECWC will be linked to sexual interests in children (H2.1). We also assumed ECWC to be a stronger predictor of sexual interests in children than empathy (H2.2). Further, we expected a moderating effect of child-related professions in these relationships (H2.3).

## Method

### Design and procedure

The study was administered online via two virtual laboratories at the FernUniversität in Hagen (Hagen University), Germany (August 2020 to February 2021) and MSH – Medical School Hamburg, Germany (March to June 2022) using the online survey tool called Unipark – Enterprise Feedback Suite (EFS) Survey (previously Questback GmbH, currently Tivian IX GmbH) and Inquisit 6 Web (Millisecond LLC). Before the beginning of the data collection, each survey was tested on students’ volunteers regarding the feasibility and technical functionality.

We distributed the open and voluntary study URL among people working with children through social contacts, through social media, as well as to counseling centers and help organizations for people with pedophilic interests. The current sample thus should be considered as a convenience sample. In order to ensure full anonymity and decrease potential reservations participating in this study, in which deviant sexual interests are investigated, we did not record from where the participants obtained access to the study. Both of the IAT were embedded via a URL to the Inquisit experiment within the EFS survey, thus, there was only one URL for participating in the study. EFS Survey does not allow multiple participation via URL by default. We cannot rule out if participants intentionally deleted the cookies on their devices to participate multiple times. All the materials including the study advertisement, e-mails sent out to the organizations, informed consent, survey, the IAT stimuli, and the debriefing in German language can be provided upon request. The informed consent covered the information on the purpose and the length of study, data managements and storage information, contact information of the project management (first author) and the students who wrote their masters’ theses in the project. Students were offered course credit for their participation. Non-student participants had the option to win a 15 EUR Amazon voucher.

Participants were asked to complete the C-ECWC (1 page, 12 items), both of the IATs (1 page each for 138 trials in total), the questionnaires for empathy (1 page, 28 items) and sexual interests (1 page, 20 items), and to provide some demographic information (1 page, 8 items), which took approximately 45 min in total. It was not possible for the participants to review and/or change their answers after proceeding to the next page. We neither randomized the survey items nor used adaptive questioning. All the items in the questionnaire were programmed as mandatory (i.e., the participants must have submitted their responses to proceed), except for the items covering sexual interests (i.e., the participants could ignore the warning regarding the incomplete response to proceed).

### Participants

#### Sample size calculation

##### Hypothesis 1.1 to 1.3 and 2.1

G*Power 3.1.9.2. indicated that a minimum sample size of 192 was needed for a Steiger’s *z*-test with two dependent correlations (ab = 0.3, ac = 0.5) with a common index (bc = 0.3) to achieve 85% statistical power and a 5% alpha error rate. Considering 10% outliers, 211 participants were targeted.

##### Hypothesis 2.2 and 2.3

Since G*Power does not provide an exact sample size estimation for moderation analyses, we used a power analysis for an ANCOVA for the comparison of the predictive power of ECWC and empathy regarding sexual interests in children (H2.2), considering that we have 2 groups (H2.3) and 7 covariates (3 ECWC subscales and 4 empathy subscales) in the design. G*Power indicated that a minimum sample size of 264 was needed to achieve a medium effect size of *f* = 0.25 with 85% statistical power and a 5% alpha error rate. Considering 10% outliers or possible irresponsible responding, 291 participants were targeted. Since H2 required a larger sample size, we planned to recruit 291 participants for the whole project.

#### The current sample

**Fig. 1:**
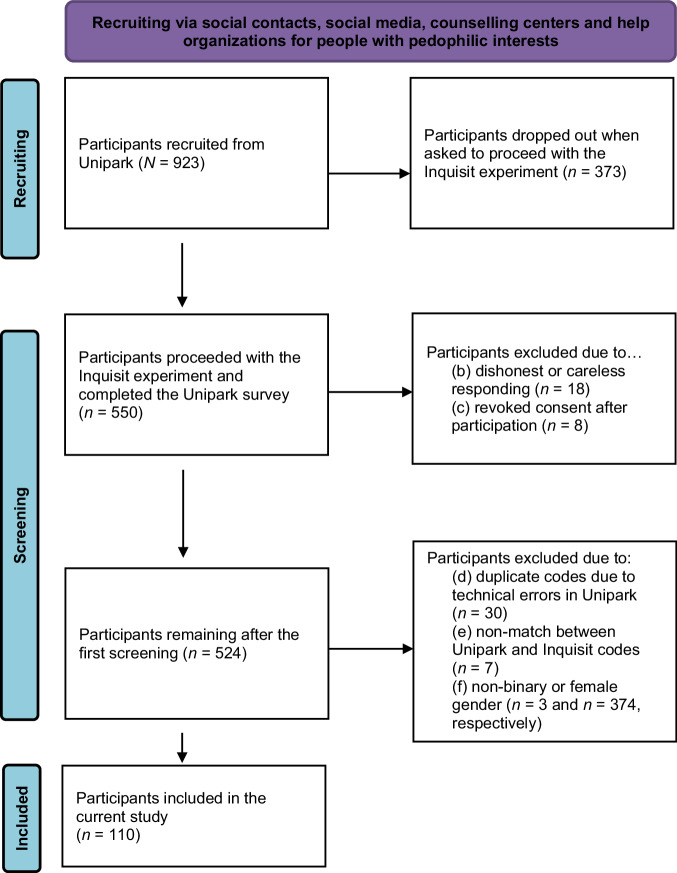
Flow diagram of the recruitment and screening of the participants.

In total, 923 participants started the survey. Cases were excluded from the analyses if one of the following criteria was met: (a) incomplete data set due to drop out – whereas most of the participants dropped out when asked to proceed with the Inquisit experiment – (*n* = 373), (b) dishonest or careless responding (*n* = 18; these participants failed the attention check [by reporting that they were allergic to water] or they did not participate seriously), (c) revoked consent after participation (*n* = 8), (d) duplicate codes due to technical errors in Unipark (*n* = 30), (e) non-match between Unipark and Inquisit codes (*n* = 7), and (f) non-binary or female gender (*n* = 3 and *n* = 374, respectively). Since most of the items were mandatory in the survey and the participants could have been completed the studies with leaving questionnaire items blank, we can only provide the completeness rate, which was calculated by the participants remaining after using the exclusion criteria (a) to (c) [*n* = 524] divided by the number of participants who started the survey by giving their informed consent (*N* = 923): 56.8%. The screening and exclusion process is demonstrated in a flow chart (Fig. 1).

The final sample included 110 male participants, age ranging from 18 to 68 (*M* = 31.3*, SD* = 11.1) years. The majority of participants was well educated, with 70 (63.6%) individuals having completed secondary and 40 (36.4%) tertiary education. Sixty-six participants were students (60.0%) and 100 had a German-speaking cultural background (90.9%). Fifty-six point four percent responded that they do not work with children (*n* = 62) and 43.6% that they do, with daily (12.7%), weekly (16.4%), monthly (4.5%), and irregular contacts (10.0%). The dataset it available on: https://osf.io/2sf74/.

### Measures

#### Implicit Association Tests

We used the Evaluation and Identification IATs designed by McPhail et al. [43] using the Inquisit template written by Krüppel et al. [44]. The Identification IAT measured the component of the ECWC in which individuals associate themselves more strongly with children than adults, therefore reacting faster when the categories CHILD and SELF share one response key (i.e., compatible combination) compared to the combination of ADULT and SELF (i.e., incompatible combination). In the Evaluation IAT, more positive attitudes towards children than adults was measured. Individuals would react faster when CHILD and POSITIVE are assigned to one response key (i.e., compatible combination) compared to ADULT and POSITIVE. (i.e., incompatible combination). Picture-stimuli representing the target categories CHILD (e.g., faces of a girl or a boy) and ADULT (e.g., faces of a woman or a man) were presented on the screen, each cross-paired with words from either of the categories SELF (e.g., me, mine, myself) and OTHERS (e.g., she, him, theirs) in the Identification IAT, as well as POSITIVE (e.g., happy, rainbow, smile) and NEGATIVE (e.g., poison, cancer, sadness) in the Evaluation IAT. We combined both IATs in one experiment. Each IAT consisted of seven blocks in the following order: two simple categorization task blocks (20 trials each), one practice (4 trials) and one test block of the compatible combination of categories (40 trials), another simple categorization task block (10 trials), followed by one practice (4 trials) and one test block of the incompatible combination of categories (40 trials).

D scores for each IAT were computed separately by subtracting the average latencies in compatible blocks from those in incompatible blocks, and dividing these differences by the pooled standard deviation across both blocks. As same as McPhail et al. [43] described, we also computed the D score using only trials with response latencies between 300 ms or longer and 10,000 ms or shorter, and only for participants who reacted correctly in 80% or more of the trial in each block. We also applied a penalty for error trials replacing the latencies with the participants’ block mean latencies plus 600 ms. McPhail et al. [43] reported Cronbach’s *α* of 0.55 for the Identification and 0.75 for the Evaluation IAT. The α values were 0.71 and 0.76 in the current sample, respectively. The split-half-reliability of the Identification and Evaluation IATs was determined using the odd-even method, *r*_*tt*_ = 0.61*, M*_*diff*_ = 0.18*, t*(485) = 11.81*, d* = 0.35, and *r*_*tt*_ = 0.73*, M*_*diff*_ = 0.09*, t*(485) = 5.90*, d* = 0.32, respectively (*p* < 0.001). We computed separate scores for the odd (d1) and even (d2) trials of the compatible and incompatible blocks in each IAT and correlated these scores within the each IAT to test the split-half reliability.

#### Explicit measures

The psychometric values of the instruments are presented in Table 1. The mean values refer to the average item scores to provide a better understanding of the position on the likert-scale.

**Table 1:** Psychometric scores of the explicit measures.

	*M*	*SD*	α	*MIC*	*W* (*p*)	*Possible item scores*
C-ECWC	2.01	0.37	0.69	0.18	0.988 (0.428)	1 to 4
C-ECWC CS	1.73	0.49	0.70	0.31	956 (0.001)	
C-ECWC RA	2.66	0.52	0.29	0.10	0.917 (<0.001)	
C-ECWC FP	1.65	0.57	0.54	0.36	0.932 (<0.001)	
IRI	3.25	0.39	0.82	0.14	0.976 (0.041)	1 to 5
IRI FS	3.23	0.66	0.72	0.27	0.983 (0.172)	
IRI EC	3.68	0.54	0.74	0.29	0.980 (0.090)	
IRI PD	3.64	0.65	0.78	0.35	0.975 (0.039)	
IRI PT	3.62	0.57	0.78	0.35	0.983 (0.174)	
SIC	1.20	0.66	0.97	0.67	0.325 (<0.001)	1 to 7

*Notes*. *M* Mean item scores, *SD* Standard Deviation, α Cronbach’s Alpha, *MIC* Mean Inter-item Correlation, *W* Shapiro-Wilk Test, *p* *p*-value, *C-ECWC* Cognitive and Emotional Congruence With Children scale, *CS* Childlike Sense of self subscale, *RΑ* Romantic Attraction to children subscale, *FP* Friendship and Positive relationships with children subscale, *IRI* Interpersonal Reactivity Index, *FS* Fantasy subscale, *EC* Empathic Concern subscale, *PD* Personal Distress subscale, *PT* Perspective Taking subscale, *SIC* Sexual Interests Cardsort, Listwise *N* = 110.

##### Cognitive and emotional congruence with children scale

The C-ECWC is a questionnaire with 12 items. The response options are on a 4-point Likert scale ranging from 1 (*strongly disagree*) to 4 (*strongly agree*), with a total score ranging from 12 to 48. The questionnaire was translated and back-translated from English to German. Paquette and McPhail [33] found a three-factor solution with the following subscales: Childlike sense of self items (e.g., “I feel like I am at the same emotional level as children”), Friendship and positive relationships with children (e.g., “Friendships with children is more important than having sex with them.”), and Romantic attraction to children (e.g., “It is the children’s beauty and purity that attract me.”). The internal consistency of the total score (Cronbach’s α = 0.69) was considerably lower compared to Paquette and McPhail [33] (α = 0.895*, n* = 218). The reliability of the subscales varied strongly from Childlike Sense of Self (α = 0.70) being acceptable to Friendship and Positive Relationships with Children (α = 0.54), and Romantic Attraction to Children (α = 0.29) being unacceptable. We also conducted a confirmatory factor analyses applying oblique rotation based on the WLSMV estimator for polychoric correlations with the lavaan package in *R*-Studio (Version 2022.0t.1.554) for the *R*-Version 4.2.0. The model fit was not satisfactory (*χ*^2^(51) = 136.84*, p* < 0.001, Comparative Fit Index [CFI] = 0.87, Root Mean Square Error of Approximation [RMSEA] = 0.12, Standardized Root Mean square Residual [SRMR] = 0.12).

##### Interpersonal reactivity index

Empathy was assessed with the German version of the Interpersonal Reactivity Index (IRI; [45]), a 28-item questionnaire with a 5-point Likert scale ranging from 1 (*does not describe me at all*) to 5 (*describes me very well*). The IRI has four subscales with 7 items each: Perspective Taking (e.g., “I try to look at everybody’s side of a disagreement before I make a decision.”), Fantasy (e.g., “I really get involved with the feelings of the characters in a novel.”), Empathic Concern (e.g., “I would describe myself as a pretty soft-hearted person.”), and Personal Distress (e.g., “In emergency situations, I feel apprehensive and ill-at-ease”). The reliability of the overall scale (α = 0.82) was high and higher than that of the four subscales Fantasy (α = 0.72), Emotional Competence (α = 0.74), and Personal Distress (α = 0.78), and Perspective Taking (α = 0.78) with 7 items each.

##### Sexual interest cardsort

We translated and back-translated the Sexual Interests in Children (intra- and extrafamilial boys and girls) subscale (20 items) of the Sexual Interest Cardsort (SIC; [46]) for the present study, which measures various types of deviant sexual interests. The items of the SIC questionnaire were rated on a 7-point Likert scale ranging from 1 (*extremely repulsive*) to 7 (*extremely interesting*). The Items of the SIC entail rather graphic wordings, e.g., “My dick is between the legs of a young boy”. Thus, we changed the following words into less disturbing words: Cock to penis, boner to stiff penis, fuck to touch, pussy to vagina and suck to kiss, e.g., “I’m having an erection. My penis is between the legs of an eight-year-old girl”. In addition, an introductory text asked the participants to imagine that the statements were made by someone else in order to divert the context from themselves and minimize the level of disturbance as well as drop-outs. In a sample of 371 male child sexual offenders, the internal consistency of the original subscales was excellent, ranging from *α* = 0.94 to *α* = 0.97 [46]. The study by Paquette and McPhail [33] demonstrated similar internal consistency, *α* = 0.95 to *α* = 0.97. The SIC subscales correlated positively with clinically assessed sexual interest in children, supporting convergent validity (*r* = 0.34 to *r* = 0.76, all *p* < 0.001). The Cronbach’s alpha was very high at 0.97 in the current study. It should be mentioned that 61.8% of the participants (*n* = 68) responded to all items with 1 (*extremely repulsive*). Six participants (5.5%) answered at least one item with at least 5 (*somewhat interesting*). Only two participants (1.8%) showed a mean value over 4 (*neutral*), one over 6 (*very interesting*).

### Statistical analyses

We performed correlation analyses to examine the relationships between implicit ECWC, explicit ECWC (H1.1), empathy (H1.2), and sexual interests in children (H2.1) in IBM SPSS Statistics (Version 28). Since Shapiro-Wilk tests showed that the distribution of the C-ECWC subscale scores, the IRI total scores, the IRI Personal Distress, and the SIC scores departed significantly from normality (*p* < 0.05), we ran both non-parametric Spearman’s and parametric Pearson’s correlations. The significant results were identical in both of the analyses except for the association between the C-ECWC Childlike Sense of Self subscale and the IRI Personal Distress as well as Perspective Taking subscale. Since these two associations were not related to our hypotheses, we proceeded with further parametric tests for H2. Please see results section for the comparison. The overview on the Spearman’s correlation analyses is provided as a supplementary material: Table S1 (https://osf.io/2sf74/).

Potential differences between dependent sample correlations (H1.3) were analyzed using Steiger’s pattern test, in which the covariance of the matrix of inter-correlations is considered. The differences between the dependent correlations were presented with Cohen’s *q* (0.1 < *q* < 0.3: small effect; 0.3 < *q* < 0.5: moderate effect; *q* > 0.5: large effect). Steiger’s pattern test was performed using the package *cocor* and the function used in the study by Yoon et al. [47] for *R* (Version 4.2.0).

To compare ECWC and empathy in their performance predicting sexual interests in children (H2.2) and to test the moderating effect of child-related jobs (H2.3), we utilized multiple linear regression analyses and the PROCESS Macro (Version 4.1) for SPSS.

## Results

### RQ1: validity of the implicit and explicit measures of ECWC

We assumed that the implicit and explicit measures of ECWC would be positively correlated (H1.1). In contrast to our hypothesis, only the Evaluation IAT was significantly positively correlated with the C-ECWC Childlike Sense of Self subscale (*r* = 0.17*, p* = 0.039). There was no significant correlation between the Identification IAT and any of the C-ECWC scores.

Regarding the relationship between both of the IATs and empathy explicitly measured with the IRI (H1.2), the Identification IAT did not show any significant link to the IRI. The Evaluation IAT showed a significant correlation with the IRI total (*r* = 0.18*, p* = 0.028) and Personal Distress subscale scores (*r* = 0.22*, p* = 0.012). The inter-correlations of the measures are demonstrated in Table 2.

**Table 2:** Overview on the Pearson’s correlations of all measures.

	(1)	(2)	(3)	(4)	(5)	(6)	(7)	(8)	(9)	(10)	(11)
(1) C-ECWC Total											
(2) C-ECWC CS	0.803***										
(3) C-ECWC RA	0.552***	0.186*									
(4) C-ECWC FP	0.730***	0.375***	0.141								
(5) IRI Total	0.112	0.141	0.027	0.049							
(6) IRI FS	0.031	0.038	0.106	−0.066	0.757***						
(7) IRI EC	0.072	0.090	−0.068	0.104	0.661***	0.245**					
(8) IRI PD	0.145	0.199*	−0.010	0.082	0.597***	0.449***	0.087				
(9) IRI PT	0.038	0.034	0.026	0.019	0.572***	0.184*	0.488***	−0.091			
(10) SIC	0.392***	0.321***	0.404***	0.125	0.032	0.055	−0.034	0.062	−0.015		
(11) D Eval-IAT	0.086	0.170*	0.030	−0.042	0.184*	0.067	0.033	0.217*	0.151	0.122	
(12) D ID-IAT	0.114	0.120	0.070	0.043	0.073	−0.041	0.096	0.116	0.027	0.092	0.140

*Notes*. *C-ECWC* Cognitive and Emotional Congruence With Children scale, *CS* Childlike Sense of self subscale, *RΑ* Romantic Attraction to children subscale, *FP* Friendship and Positive relationships with children subscale, *IRI* Interpersonal Reactivity Index, *FS* Fantasy subscale, *EC* Empathic Concern subscale, *PD* Personal Distress subscale, *PT* Perspective Taking subscale, *SIC* Sexual Interests Cardsort, *D Eval-IAT* D Score of the Evaluation IAT, *D ID-IAT* D Score of the Identification IAT . All the coefficients are calculated using Pearson’s product-moment correlations analyses. Listwise *n* = 109;*p < 0.05, **p < 0.01, ***p < 0.001.

Also contradicting our expectations that the implicit and explicit ECWC would be more strongly correlated than the implicit ECWC and the explicit empathy measured with the IRI, the correlations did not differ from each other (H1.3). Since only the Evaluation IAT showed significant correlations with the C-ECWC Childlike Sense of Self subscale as well as the IRI total and Personal Distress scores, we compared these correlations to each other. The correlation of the Evaluation IAT with the C-ECWC Childlike Sense of Self subscale was neither larger than the one with the IRI total scores *z* = 0.032*, p* = 0.487*, χ*^2^(1, 109) = 0.001*, p* = 0.973, nor than the one with the IRI Personal Distress scores *z* = 0.278*, p* = 0.390*, χ*^2^(1, 109) = 0.078*, p* = 0.779.

### RQ2: construct validity of ECWC

In the second step, we tested the construct validity of ECWC using external criteria. The overview of the correlation analyses as well as of the SIC regressed on the C-ECWC and the IRI is provided in Table 2 and Table 3, respectively. H2.1 referred to the positive relationship between ECWC and sexual interests in children. Whereas none of the IAT scores was related to the SIC scores (Evaluation IAT: *r* = 0.12*, p* = 0.104, β = 0.06*, p* = 0.470 and Identification IAT *r* = 0.09*, p* = 0.170, β = 0.03*, p* = 0.741), the C-ECWC scores were significantly related to the SIC (H2.2; all *p* < 0.001): Total scores (*r* = 0.39, β = 0.39, both *p* < 0.001), Childlike sense of self (*r* = 0.32*, p* < 0.001, β = 0.25*, p* = 0.012), and Romantic attraction to children (*r* = 0.40, β = 0.36, both *p* < 0.001), except for Friendship and Positive relationships with children (*r* = 0.13*, p* = 0.098, β = *−*0.01*, p* = 0.861).

**Table 3:** Summary of the regression analyses on predictors of sexual interests.

	β	*t*(*df* ^a^)	*p*	*sr*
IAT				
D Eval-IAT	0.06	0.73 (103)	0.470	0.07
D ID-IAT	0.03	0.33 (103)	0.741	0.03
C-ECWC	0.39	4.39 (107)	<0.001	0.39
C-ECWC CS	0.25	2.56 (103)	0.012	0.24
C-ECWC RA	0.36	4.03 (103)	<0.001	0.34
C-ECWC FP	−0.01	−0.18 (103)	0.861	−0.02
IRI	−0.01	−0.12 (107)	0.908	−0.01
IRI FS	0.05	0.41 (105)	0.685	0.04
IRI EC	−0.05	−0.48 (105)	0.636	−0.05
IRI PD	0.05	0.42 (105)	0.672	0.04
IRI PT	0.01	0.06 (105)	0.957	0.01

*Notes*. *β* standardized beta coefficients of multiple regression analyses, *df* degree of freedom, *sr* semi-partial correlation, *C-ECWC* Cognitive and Emotional Congruence With Children scale, *CS* Childlike Sense of self subscale, *RΑ* Romantic Attraction to children subscale, *FP* Friendship and Positive relationships with children subscale, *IRI* Interpersonal Reactivity Index, *FS* Fantasy subscale, *EC* Empathic Concern subscale, *PD* Personal Distress subscale, *PT* Perspective Taking subscale, *D Eval-IAT* D Score of the Evaluation Implicit Association Test, *D ID-IAT* D Score of the Identification Implicit Association Test . Listwise *n* = 109; ^a^To avoid multi-collinearity problems, we tested the total scores and the subscales scores separately.

H2.2 regarding the differences in predictive powers of ECWC and empathy was sustained at least partly for the C-ECWC without statistical testings, since none of the IRI scores significantly predicted the SIC scores (*r* = *−*0.03 to 0.06, β = *−*0.05 to 0.05). For the IATs, we omitted comparison analyses as well, since both IATs and the IRI were not significant in predicting the SIC scores.

The next comparison was carried out between men with and without child-related professions (H2.3). As expected, there was also moderating effects of the child-related job on the link between the C-ECWC and SIC scores. The link between the C-ECWC total and the SIC scores was moderated by the child-related job, *R*^2^ = 0.04*, F*(1, 106) = 5.02*, B* = − 0.75*, p* = 0.027. There was positive link between the C-ECWC and the SIC scores in both groups with and without child-related jobs, however, scores of men with child-related jobs showed a stronger link (*B* = 1.21*, p* < 0.001) than those without (*B* = 0.46*, p* = 0.015). This interaction effect seems to be driven by the Childlike Sense of self subscale. The relationship between the Childlike Sense of self subscale and the SIC scores was moderated by the child-related job, *R*^2^ = 0.06*, F* (1, 106) = 7.56*, B* = − 0.14*, p* = 0.007. In men with child-related jobs, there was a significant positive link between childlike self-concept and sexual interests in children (*B* = 0.17*, p* < 0.001), whereas the link was not significant in men without child-related jobs (*B* = 0.04*, p* = 0.227). The interaction between the job status and the other two C-ECWC subscales predicting the SIC scores was not significant: Friendship and Positive relationships with children (*R*^2^ = 0.00*, F* (1, 106) = 0.03*, B* = − 0.02*, p* = 0.860) and Romantic Attraction to children (*R*^2^ = 0.01*, F* (1, 106) = 1.23*, B* = − 0.08*, p* = 0.270).

## Discussion

The current study was conducted as the first part of an on-going project aiming to deepen and expand the understanding of constructs which are phenomenologically associated with pedophilic sexual interests. It is the first study implementing the IATs measuring positive attributions towards children (Evaluation IAT) and childlike self-concept (Identification IAT) developed by McPhail et al. [42] combined with the C-ECWC questionnaire developed by Paquette and McPhail [33], and also the first study testing the differential relationships between the measures with other constructs, namely empathy and sexual interests in children in a community-based male sample. We tested the psychometric properties of these procedures in men from the general population (of which only 1.8% to 5.5% reported some degree of sexual interest in children), in which we found a vast range of reliability estimates from unacceptable to acceptable levels for both of the IATs and the C-ECWC. We found a positive link between the implicit positive attribution to children and the explicit childlike self-concept. Contradicting our assumption, we did not find more plausible links, for instance, between implicit positive attitudes and explicit preference of friendship and positive relationships with children. Implicit identification with children should have been also rather correlated with the explicit identification.

Compared to the study by McPhail et al. [42], which identified small to moderate correlations in both between implicit and explicit identification with children and positive attitudes towards children in child sexual offenders, the current results did not sufficiently support the validity of both IATs. Similarities in findings regarding the implicit-explicit link were particularly regarding the link between the implicit childlike self-concept and the C-ECWC questionnaire, which was also not significant. In the offender sample, despite the very small sample size, McPhail et al. [42] found a significant link between the implicit childlike self-concept and professionally assess emotional identification with children, as well as sexual interests revealed in victimology. Although we might find similar links in an offender sample in the future, the question arises, if the difference in the findings might have resulted from the weak effects of the IAT [38], and we could not detect its link to other measures due to floor effects (see limitations below related to the online sample). Another possible explanation could be the lack of clarity regarding the measurement outcomes. A mere association between self and child(ren), for instance, can have several reasons, including stronger identification with children, but also stronger sexual interest in children, or more positive attitudes towards children. A further important question that remains unresolved is whether the implicit identification with children was conflated with the implicit self-concept as an adult in the present operationalization of the Identification IAT. In addition, as discussed earlier, the underlying mechanism of implicit measures is still debated. Whereas some call it implicit in the way that it captures unconscious associations, others use the term as a synonym for automatic. We thus can neither be certain that the Identification IAT really captured the target concept, nor can we be certain that both IATs captured distinct concepts. It is therefore conceivable that the absence of significant effects indicates limitations of the measures rather than a lack of correspondence between the target concepts.

The scarce correlations we found could also have resulted from the lower reliability of the Identification IAT (*α* = 0.71*, r*_*tt*_ = 0.61). In addition, both of the C-ECWC subscales were unrelated to other measures: Romantic Attraction to children subscale and Friendship and Positive relationships with children subscale (*α* = 0.29 and *α* = 0.54, respectively). Moreover, the latent structure of the C-ECWC questionnaire was less satisfactory in the present sample consisting of young non-offending males, compared to the initial study by Paquette and McPhail [33] consisting of both community and offender samples.

Aligning with the previous finding that found ECWC as an independent construct [42], our results also suggest that ECWC is generally unrelated to empathy. We nevertheless found a specific correlation between childlike self-identification and personal distress in terms of anxious and unease feelings in tense interpersonal settings [45]. This link can be interpreted against the backdrop of Finkelhor [25]’s predisposition theory, which suggested that men with pedophilia are inhibited in interacting with adults due to their deficient emotional processing. This could lead to negative emotions, such as loneliness. Research has suggested emotional immaturity and distorted emotional processing of men with pedophilia [21–23]. Since these variables were not normally distributed and we found this link only in the parametric test, this interpretation remains speculative on this stage.

Baring these limitations in the first step, we proceeded with the second step testing the associations of the ECWC measures with sexual interests in children compared to an empathy measure, as well as in men with or without contacts with children in their occupational contexts. We found favorable results especially for the C-ECWC questionnaire. With the exception of the subscale Friendship and Positive relationships with children, the other two subscales measuring childlike self-concept and romantic attachment with children – hence the total scores – predicted sexual interests in children with moderate effect sizes. Neither the positive associations and childlike self-identifications detected in the IATs nor empathy predicted increases in deviant sexual interests. Further, the association between ECWC and sexual interests in children were stronger in men working with children than those who do not. These results are in accordance with previous findings that ECWC is a possible indicator of increased sexual interests in children [26–28]. Moreover, the difference between men with and without child-related jobs aligns also with Finkelhor [25]’s assumption. It seems that men with both increased ECWC and deviant sexual interests in children seek more contact with children. The fact that the level of sexual interests was very low in this sample and the study was conducted using a cross-sectional design, mandates cautious interpretation of this finding.

### Limitations

One of the major limitations of the current study is that only 1.8% to 5.5% of our sample reported some degree of sexual interest in children. In addition, the study was conducted in an online sample, in which the education level was fairly high. Sexual interests in children in general population are rarely prevalent compared to the offender population, which was also the reason why we distributed the study link to counseling centers and help organizations for people with pedophilic interests. This floor effect has probably decreased the chance to identify an actual link between the measures in the current sample. As the next step of the project, thus, we are planning to cross-validate these findings in an offender sample.

Another important limitation is that we changed the wordings in the SIC to avoid drop-outs. Instructing participants to imagine that someone else is reporting deviant behavior – even if we kept the first-person sentences in the questionnaire itself –, might have distorted the actual prevalence of deviant sexual interests in the current sample. The measures should rather be implemented in laboratory settings in future studies with an informed consent being communicated directly by the researchers rather than solely in a written text.

Another sample-related issue is that we did not have enough participants in each group to detect smaller effects. Considering that ECWC is more strongly related to sexual interests in men working with children, yet the effect sizes were small, we would have needed at least 264 individuals to detect the observed effect size with 5% alpha-error probability and 85% power. We had 110 individuals in total, 48 individuals in the respective group resulting in 40% power. Thus, there is a 60% possibility that we have incorrectly rejected the false null hypothesis and failed to detect actual link between the variables.

Lastly, the current study only validated the implicit measures using self-reported explicit measures. To our knowledge, there is still no other study than the one by McPhail et al. [42], which utilized various data sources. Since the survey was online and we did not have experts’ ratings on sexual deviance, the weaker links we found might have resulted from the different modes of assessment for socially undesirable constructs. As IATs are commonly perceived as less affected by social desirability [18], faking might account for the differences in measurement outcomes. Our next goal will be therefore to expand constructs – further measures of ECWC, empathy, and cognitive distortions – as well as mode of assessments – self-reports and observer-ratings – in a larger sample including people who have committed sexual offenses against children. Future research should also investigate individuals who have sexual interests in children but have not sexually abused children and to expand the target to females with sexual offenses against or sexual interests in children.

## Conclusion

The present study demonstrates the first step of an on-going project exploring differential links within the nomological net of constructs related to pedophilia. We examined the psychometric properties of the implicit and explicit measures of ECWC in a sample of men from the general population who mostly did not indicate a sexual interest in children and found heterogeneous reliability values and weak links between the measures. We established nevertheless a clearer link between explicitly reported ECWC and sexual interests, whereas empathy was not linked to ECWC. The links were also moderated by child-related occupations, which implicates that men working with children reveal stronger connections between their ECWC and sexual interests in children.

Further studies need to replicate and expand the present study design utilizing the measures in offender samples in order to incorporate these findings into the full network of pedophilia-related constructs. Despite the somewhat unfavorable findings for the IAT, it should be mentioned that C-ECWC scales yield valuable information predicting sexual interests in children, especially in men working with children. Future studies in offender samples are warranted to resolve these questions regarding the reliability and validity of the measures for ECWC.

## Data Availability

Data of the current study is available at: https://osf.io/2sf74/.
